# Knowledge, Attitudes, and Practices about Zika among a University Community Located in an Endemic Zone in Mexico

**DOI:** 10.3390/ijerph15112548

**Published:** 2018-11-14

**Authors:** Vianey G. Argüelles-Nava, María T. Alvarez-Bañuelos, Daniel Córdoba-Suárez, Clara L. Sampieri, María C. Ortiz-León, Gabriel Riande-Juárez, Hilda Montero

**Affiliations:** 1 Instituto de Salud Pública, Universidad Veracruzana, Xalapa 91190, Veracruz, Mexico; varguelles@uv.mx (V.G.A.-N.); talvarez@uv.mx (M.T.A.-B.); csampieri@uv.mx (C.L.S.); cortiz@uv.mx (M.C.O.-L.); griande@uv.mx (G.R.-J.); 2 Licenciatura en Ciencias y Técnicas Estadísticas, Universidad Veracruzana, Xalapa 91000, Veracruz, Mexico; danielcordoba.suarez@gmail.com

**Keywords:** Zika virus, disease prevention, knowledge, attitudes and practices towards health, health communication, university students, university workers, sexual health, infection

## Abstract

To assess the knowledge, attitudes, and practices about the Zika virus in both students and workers at the University of Veracruz, an online survey was conducted. The participants were divided into two groups: one according to sex, the other according to whether they were workers or students. Their answers were classified into knowledge, attitudes, and practices and they were rated as low, medium, and high. The results showed that knowledge about Zika prevailing among the university population is considered as medium in 79.4% of the study population. Most respondents know that the mosquito spreads the Zika virus (98.8%) and the clinical characteristics, while sexual transmission by the virus is little known (36.85%). Both the univariate analysis (OR (CI5) 0.227 (0.070–0.735), *p* = 0.013] and multivariate analysis (OR (CI95) 0.234 (0.071–778), *p* = 0.018] showed that belonging to the health sciences area is related to having a greater knowledge about Zika. Despite the existing knowledge, a low level of prevention practices prevails in the whole community (55%). A medium level of knowledge about Zika prevailed, while proper implementation of preventive measures for Zika is low, despite the fact that the state of Veracruz—the place where the University is located—is an endemic area.

## 1. Introduction

The Zika virus has a genome of ribonucleic acid and belongs to the genus Flavivirus. It is an arbovirus because it uses certain arthropods as vectors, such as the *Aedes aegypti* mosquito [1]. Unlike other arboviruses, such as dengue virus and Chikungunya virus, Zika virus can be transmitted either sexually or vertically during pregnancy from mother to child [2,3]. Although an absence of symptoms has been reported in most of the subjects infected by this virus [4], they still have the potential to transmit the virus sexually, as well as to the vectors. Individuals with symptoms of Zika usually have fever, arthralgia, exanthem, headache, and conjunctivitis during the period called the acute phase, which lasts about a week [5]. Zika disease is usually tolerable: most patients say they have a simple eruptive skin rash. However, as a neurotropic virus, it has been proven that it can cause neurological harm in adults by producing the Guillain–Barré syndrome [6].

Of great interest recently for public health has been the congenital syndrome caused by Zika virus from the vertical transmission of the virus from mother to child. This syndrome includes microcephaly, subcortical and intraocular calcifications, arthrogryposis and a variety of problems: visual, hearing, cognitive and developmental [7,8]. It has been proposed that the effects of the Zika virus on the neonate are a consequence of its ability to infect brain cells [9].

More than 2000 neonates with microcephaly caused by Zika virus have been reported worldwide [10]. In addition, the number of newborns with congenital abnormalities that may affect learning, hearing, and vision, among other sequelae, has not been estimated yet. In Brazil, some women have decided not to get pregnant because of the impact that has been caused by the Zika virus [11].

According to the various health problems caused by the Zika virus and its modes of transmission, society plays a key role in preventing this infection. In this sense, health institutions inform people about how to prevent Zika; also, the social networks have an important role. However, the information provided by social networks is not always accurate [12]. The World Health Organization (WHO) designed a survey called Knowledge, Attitudes and Practices: Zika Viral Disease and Its Possible Complications (KAP) [13] in order to explore these aspects among the general population so that the health authorities may design appropriate strategies to control this disease.

Zika disease is considered endemic to Mexico, mainly in states where the *Aedes aegypti* mosquito is found. The Centers for Disease Control and Prevention (CDC) (Atlanta, GA, USA) have classified the high-risk countries to alert people who travel to these places [14]. Pregnant women are advised not to travel to risk areas; if the trip is absolutely necessary, the use of safe repellents is recommended [15]. All people should take the appropriate measures to avoid being bitten by the mosquito or engaging in unsafe sexual practices [14].

Since the Zika virus has started to circulate recently, the amount of knowledge that people have about Zika disease and the virus is unknown. This is the reason why this study aims to assess the knowledge, attitudes, and practices towards Zika among the population living in Veracruz, which is one of the most arbovirosis-affected areas in Mexico [16], specifically among students and workers of the University of Veracruz.

## 2. Materials and Methods

### 2.1. Tools

A questionnaire was designed according to the WHO’s resource folder for KAP surveys to explore the Zika virus infection and its possible complications. The questionnaire was reduced to 35 multiple-choice questions. For some questions, it was possible to choose more than one answer [13]. Then, it was adapted to Mexican Spanish by a multidisciplinary team of researchers working for the Public Health Institute, attached to the University of Veracruz. This multidisciplinary team included four questions on topics not considered by the WHO’s resource folder: two questions to identify knowledge about the specific capacity of some insect repellents against the mosquito that transmits the Zika virus; one question to determine the preventive practices that people would conduct in case they were at risk of infection in their communities; and another question to identify knowledge about the availability of a vaccine against the virus. The final questionnaire consisted of 48 questions that were distributed as follows: (a) demographic characteristics (9 questions); (b) knowledge about Zika (21 questions); (c) attitudes towards Zika (7 questions), and (d) practices towards Zika (11 questions).

### 2.2. Data Collection

The information was collected from 10 October to 9 November 2016. All workers and students were invited to participate by mass communications from the University of Veracruz: Facebook, Twitter, e-mail, top-rated radio programs, and a weekly printed and digital university newspaper. 

The Survey Monkey^®^ online survey was used. Access was limited to workers and students; both had to use their institutional e-mail account. Before answering the survey, their informed consent about the data collection protocol, background, and purpose of the survey, as well as the usage and confidentiality of the information was recorded. Voluntary participation was expressly declared. Thus, both informed consent and voluntary participation were the requirements to access the questionnaire. The study was approved by the Technical Council of the Public Health Institute, belonging to the University of Veracruz. Accessing the electronic questionnaire and language comprehension were verified prior to its dissemination.

### 2.3. Scoring of Responses

The scores obtained for knowledge and practice were calculated separately. Questions answered correctly were worth 1 point; otherwise, they were worth 0. The maximum scores were 30 points for knowledge and 15 for practice. Later, these scores were re-coded—for knowledge: 0 to 10, low; 11 to 20, medium; and 21 to 30, high; for practice: 0 to 5, low; 6 to 10, medium; and 11 to 15, high.

### 2.4. Information Analysis

A database was created using the Statistical Package for the Social Sciences (SPSS) software, version 18 (IBM SPSS Inc., Armonk, NY, USA), with data from the participants. Respondents who answered less than 50% of the questionnaire were excluded. Hence, the eligible participants totaled 749. Descriptive statistical measures were calculated for each multiple-choice question. The open questions were analyzed by H.M. and M.C.O.L., who classified it according to what was expressed by the participants; in case of non-coincidence in any of the categories, this was agreed between the two researchers. The terms of this classification were represented in a cloud of words using the online software Word Art. To facilitate the comparative analysis of the KAPs, some variables were re-categorized and the proportions were compared using the X^2^ test. To evaluate which characteristics were associated with low knowledge against the medium or high scores of the participants, both a univariate and multivariate analysis were performed using a logistic regression model.

## 3. Results

### 3.1. Population Characteristics

Out of the total number of 749 surveys, 503 (67%) were women, while 246 (33%) were men. The age group with the highest response rate was that of 20 to 24 years (37%), followed by young people ranging from 15 to 19 years (35%). Most respondents were students, with a total number of 584 (78%), while the rest were workers from different units of the University of Veracruz.

High school was the highest level of education that was most frequent in this study (48%), because many respondents who participated were undergraduate students. The survey had a greater response from the Health Sciences area (16%), followed by the economic administrative area (11%). As regards the distribution of participation in the survey among the five regions of the University, the region of Veracruz prevailed (24%) (Table 1).

### 3.2. Knowledge about Zika, Preventive Attitudes, and Practices

According to the survey proposed by the WHO, some variables were considered to study population. They were divided into knowledge (a set of understandings), attitudes (a way of being or a position), and practices (the observable actions of an individual) about Zika. The variables that were found to have statistical significance are shown in Table 2.

Regarding attitudes, 50.6% of people said they did not have enough information about Zika, while 57.01% said the information they have received about this disease is rarely clear.

As regards knowledge about Zika and prevention practices, the pertinent answers were assessed by assigning a scale so that knowledge was classified as low, medium, and high. The results of the analysis show that most of the population has a medium level of knowledge about the disease and its transmission (79.4%). Meanwhile, a low level was most frequent for practices (55%) (Table 3). Both the univariate analysis (OR (CI95) 0.227 (0.070–0.735), *p* = 0.013] and multivariate analysis (OR (CI95) 0.234 (0.071–778), *p* = 0.018] showed that being a part of the health sciences area is related to having a greater knowledge about Zika (Table 3).

Correlation analyses between knowledge and practices show a discrepancy, suggesting that although people have knowledge about the risk of Zika infection, proper preventive measures are not taken (*p* < 0.001) (Table 4 and Table 5).

### 3.3. Gaps in Knowledge about Zika and Prevention Measures

**Figure 1:**
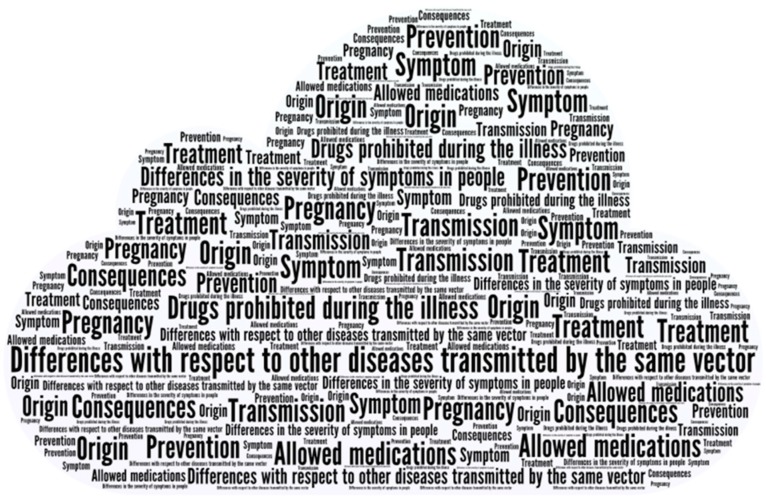
Word cloud for the open question: “What aspects do you not understand or are confusing for you about Zika?”.

In order to learn about concerns and points that are of interest to the study population, at the end of the survey, we asked this question: “What aspects do you not understand or are confusing for you about Zika?” In this regard, the responses were diverse and grouped into the following topics: treatment, consequences, transmission, and prevention of mosquito bites. Regarding treatment, people have a few queries about what medications they can or cannot take during the infection and whether there is a specific diet. As regards consequences, people have a few queries about sequelae of the infection and how to detect them. With regard to the transmission and prevention of mosquito bites, the questions were about resistance to larvicide products, whether the mosquito itself can transmit the disease, and about all of the transmission modes (Figure 1). The ten most frequently encountered answers were (1) differences with respect to other diseases transmitted by the same vector, (2) treatment, (3) transmission, (4) symptoms, (5) effect on pregnancy, (6) origin, (7) consequences, (8) drugs prohibited during the illness, (9) drugs allowed, and (10) prevention.

## 4. Discussion

The Zika virus started to circulate in Mexico recently [17]. It is a virus with particular characteristics due to the wide range of diseases it can cause [5,6,7,8]. In addition, it can be transmitted in three different ways [9,10,18]. When the epidemic started, WHO alerted the world by providing the media with information related to Zika. However, a few months later, such information was removed, with Zika becoming a virus of importance to reproductive health [19].

Despite the advertising received by this public health emergency, which was of local, national, and international interest, especially before and during the month of November 2016, this study showed that the members of the university community still possess incorrect information. However, in general terms, the results showed that the level of knowledge about Zika that the student community possesses can be considered as medium (79.4%), which is consistent with living in an endemic area. The fact that the population possesses a medium level of knowledge is not only because they are part of the university population, since studies conducted with similar populations have shown a low level of knowledge about the same disease [20]. The symptoms are well identified by the majority: exanthem is the hallmark of Zika [21], which was well identified by 82.64% of the study population. Much of the knowledge gained by the student population is because they have met someone who has been infected like their family (44.73%), friends or neighbors (35.78%). They also pointed out that television (61.82%) and the Internet (58.61%) are their knowledge sources. Health authorities influenced knowledge to a lesser extent, showing that institutions should be greatly involved when disseminating information and educating people in Zika to prevent and identify possible cases. There is an at-risk population that does not have access to information in the same way as a student or worker of a university community. Also, the same information could be more difficult to understand for that population. This population should be addressed by the authorities with specific strategies to help prevent infection, which is an important task in terms of health.

Regarding knowledge of transmission, most people know that the mosquito transmits Zika (98.8%), while only 36.85% know about sexual transmission. The group of workers is well informed about sexual transmission. This transmission mode is not common for arboviruses; perhaps because of this, many people continue to ignore this transmission mode. This lack of knowledge is consistent with other endemic [20,22,23] and non-endemic [24] populations and, also, with the decreased use of condoms to prevent Zika in 83% of the study population from Veracruz. It is mandatory that, at least in endemic areas, protected sexual contact should be practiced during pregnancy. Thus, this recommendation should be resumed by the authorities.

The WHO package for designing and conducting KAP surveys on both Zika virus and its possible complications is a useful resource for these surveys. However, it does not consider exploring how specific repellents are used. Hence, in our study, we added an additional question in order to explore whether people know what the most proven effective repellents are. We found out that the vast majority of respondents (71.43%) do not know which ones are the most effective. Therefore, we consider that this aspect should be included in all of the campaigns to prevent Zika.

The teratogenicity linked to the Zika virus is known by most people. However, they attribute various consequences to the infection while the fetus is in development. Less than 1% said that this infection does not cause any consequences in pregnancy. This topic has been explored in other populations where there is a full understanding [23,25]. This is very important given the ecological, epidemiological, and socioeconomic conditions that characterize Mexico, particularly the State of Veracruz, due to the distribution of vectors and the spread of viruses [26].

A part of the survey allowed the population to express their queries. Thus, we found out important aspects that may be useful when planning prevention campaigns for vector-borne diseases, in particular, and designing permanent strategies to prevent the possible transmission of the Zika virus. Although a medium level of knowledge was found, the university community says that they have many doubts and they are not sure whether what they “know” is correct and they feel they should have more information. Most people who responded to the survey had queries about efficient chemicals such as larvicides, where they can be screened for Zika virus, what medications to take or not to take, among other queries. Interestingly, these variables have already been proposed by other studies and have been considered as gaps that should be taken care of by the health authorities in future campaigns to educate the population and improve prevention mechanisms [27].

The results of this survey suggest that the majority reported they feel they are at risk of infection in their region. However, protection practices, for the most part, are generally not used. In this study, the reason for this was not investigated. This is a matter of concern and would be an issue for further investigation in order to establish strategies that lead to a better implementation of protective practices, because despite having proper knowledge about Zika, it is not related to preventing the infection.

The results observed in this study could be a reflection of the difficult task of dealing with the *Aedes aegypti* by endemic countries, and the transformation of knowledge, attitudes, and therefore practices for removing vectors and preventing infections. Additionally, the limited information available on the behavior of the virus in our population is a factor that causes poor understanding.

With regard to countries that are exempt from the mosquito that transmits the Zika virus, health authorities should make this population aware of the disease, especially if at-risk persons need to travel to these areas of risk, since they could import the virus through sexual transmission. Pregnant women should not travel to places of risk and if their sexual partners do, they should use a condom once they return home [15]. For the correct flow of information, the CDC makes known those regions where mosquito bite infection is likely [14]. In addition, it indicates the active principles that are effective against mosquito bites [28] and that are not dangerous for use during pregnancy [15,28].

## 5. Conclusions

Although people know about the risk of Zika infection, proper preventive measures are not taken, so Mexican health services must develop strategies to promote self-care as well as vector control because it is a teratogenic virus that will continue to circulate in the population until there is a vaccine. In addition, similar studies should be carried out in populations with lower academic levels, which tend to be more vulnerable.

## Figures and Tables

**Table 1:** Demographic characteristics of the participants.

Characteristic	General	Sex		Status	
		Women	Men	Workers	Students
	*n* = 749	*n* = 503	*n* = 246	*n* = 165	*n* = 584
Sex ^a^					
Woman	503 (67)	-	-	100 (61)	403 (69)
Man	246 (33)	-	-	65 (39)	181 (31)
Age ^b^	21 (27–19)	20 (24–19)	22 (34–19)	47 (56–38)	20 (21–19)
Age Groups ^a^					
15–19	262 (35)	193 (38)	69 (28)	0 (0)	262 (45)
20–24	277 (37)	185 (37)	92 (37)	2 (1)	275 (47)
25–39	91 (12)	52 (10)	39 (16)	49 (30	42 (7)
40 and over	119 (16)	73 (15)	46 (19)	114 (69)	5 (1)
Highest Level of Education ^a^					
High School or less	358 (48)	263 (52)	95 (39)	4 (2)	354 (61)
Student at University	241 (32)	153 (30)	88 (36)	29 (18)	212 (36)
Master’s degree	62 (8)	33 (7)	29 (12)	62 (38)	0 (0)
Specialty	13 (2)	8 (2)	5 (2)	11 (7)	2 (0)
Doctorate	59 (8)	41 (8)	18 (7)	59 (35)	0 (0)
No response	16 (2)	5 (1)	11 (4)	0 (0)	16 (3)
Academic Area ^a^					
Arts	4 (1)	2 (0)	2 (1)	2 (1)	2 (0)
Biological-agricultural	24 (2)	12 (2)	12 (5)	19 (12)	5 (1)
Health Sciences	118 (16)	91 (18)	27 (11)	44 (27)	74 (13)
Economic-Administrative	81 (11)	49 (10)	32 (13)	9 (5)	72 (12)
Humanities	28 (4)	16 (4)	12 (5)	22 (13)	6 (1)
Technical	44 (6)	19 (4)	25 (10)	31 (19)	13 (2)
Others	35 (5)	22 (4)	13 (5)	30 (18)	5 (1)
No specified	415 (55)	292 (58)	123 (50)	8 (5)	407 (70)
Region ^a^					
Coatzacoalcos-Minatitlán	73 (10)	46 (9)	27 (11)	13 (7)	60 (11)
Córdoba-Orizaba	113 (15)	88 (17)	25 (10)	24 (15)	89 (15)
Poza Rica-Tuxpan	145 (19)	96 (19)	49 (20)	16 (10)	129 (22)
Veracruz	178 (24)	114 (23)	64 (26)	31 (19)	147 (25)
Xalapa	131 (17)	72 (14)	59 (24)	80 (48)	51 (9)
No specified	109 (15)	87 (17)	22 (9)	1 (1)	108 (18)

^a^ Data are presented in *n* (%); ^b^ The variable is not distributed normally. Data are presented in median (Third quartile–first quartile).

**Table 2:** General knowledge about Zika.

Question/Response	Sex					Status				
	Women		Men		*p*	Workers		Students		*p*
	*n* = 503		*n* = 246			*n* = 165		*n* = 584		
	*n*	%	*n*	%		*n*	%	*n*	%	
**Knowledge**										
The person knows about the chances of becoming infected with Zika in the community	367	72.96	175	71.14	0.806	139	84.24	403	69.01	<0.001 *
You have met someone who has been infected with Zika	363	72.17	168	68.29	0.326	107	64.85	424	72.60	0.046 *
Ways to get Zika										
Washing with contaminated water	6	1.19	11	4.47	0.005 *	0	0.00	17	2.91	--
Sexual transmission	187	37.18	89	36.18	0.790	92	55.76	184	31.51	<0.001 *
Unhealthy environment	71	14.12	22	8.94	0.044 *	11	6.67	82	14.04	0.011 *
Signs and Symptoms of Zika										
Headache	412	81.91	207	84.15	0.448	123	74.55	486	83.22	0.012 *
Rash	429	85.29	190	77.24	0.006 *	130	78.79	489	83.73	0.139
Joint pain	409	81.31	184	74.80	0.039 *	122	73.94	471	80.65	0.061
Conjuntivitis (swollen eyes)	296	58.85	128	52.03	0.077	105	63.64	319	54.62	0.043 *
Hemorrhage/bleeding	25	4.97	21	8.54	0.001 *	7	4.24	39	6.68	0.250
Always there are Zika symptoms in infected people	306	60.83	142	57.72	0.629	86	52.12	362	61.99	<0.001 *
The person has knowledge about Zika prevention	394	78.33	204	82.93	0.329	141	85.45	457	78.25	0.007 *
Ways to prevent ZIKA										
Burning mosquito coils or lightning bonfires to repel mosquitos	79	15.71	55	22.36	0.026 *	40	24.24	94	16.10	0.016 *
Wearing clothes that cover most of the body	295	58.65	126	51.22	0.054	115	69.70	306	52.40	<0.001 *
Using condoms	139	27.63	74	30.08	0.486	68	41.21	145	24.83	<0.001 *
Abstaining from sex	43	8.55	22	8.94	0.857	13	7.88	52	8.90	0.680
Fumigation at home	369	73.36	170	69.11	0.223	104	63.03	435	74.49	0.004 *
Placing mosquito nets on windows and doors	380	75.55	172	69.92	0.100	139	84.24	413	70.72	<0.001 *
Keeping the house clean	261	51.89	127	51.63	0.946	63	38.18	325	55.65	<0.001 *
Using non contaminated water for personal care	38	7.55	15	6.10	0.465	3	1.82	50	8.56	<0.001 *
Risks a pregnant woman must face when getting infected with Zika										
Fetus may not grow or develop normally within her womb	328	65.21	153	62.20	0.419	83	50.30	398	68.15	<0.001 *
Involuntary abortion	167	33.20	86	34.96	0.633	21	12.73	232	39.73	<0.001 *
Premature birth	150	29.82	62	25.20	0.188	15	9.09	197	33.73	<0.001 *
Stillbirth	62	12.33	32	13.01	0.791	7	4.24	87	14.90	<0.001 *
The baby may be born with microcephaly	345	68.59	149	60.57	0.030 *	146	88.48	348	59.59	<0.001 *
The baby may be born with disabilities	188	37.38	88	35.77	0.669	45	27.27	231	39.55	0.004 *
Information sources on Zika										
Family	231	45.92	104	42.28	0.346	41	24.85	294	50.34	<0.001 *
Friends or neighbors	185	36.78	83	33.74	0.415	35	21.21	233	39.90	<0.001 *
Meetings with the community/community leaders	14	2.78	15	6.10	0.027 *	3	1.82	26	4.45	0.121
Door-to-door campaigns	28	5.57	15	6.10	0.769	2	1.21	41	7.02	0.005 *
Health workers at health centers	138	27.44	50	20.33	0.035 *	28	16.97	160	27.40	0.006 *
Health agents or community volunteers	64	12.72	33	13.41	0.791	10	6.06	87	14.90	0.003 *
Television	325	64.61	138	56.10	0.024 *	93	56.36	370	63.36	0.103
Written press	64	12.72	48	19.51	0.014 *	59	35.76	53	9.08	<0.001 *
Internet	285	56.66	154	62.60	0.121	118	71.52	321	54.97	<0.001 *
Social networks	199	39.56	92	37.40	0.568	53	32.12	238	40.75	0.045 *
Government ads	75	14.91	21	8.54	0.014 *	17	10.30	79	13.53	0.274
International organization	22	4.37	23	9.35	0.007 *	22	13.33	23	3.94	<0.001 *
Local/national organization	21	4.17	12	4.88	0.660	15	9.09	18	3.08	0.001 *
**Attitudes**										
Greatest concerns about Zika										
The virus can kill	210	41.75	99	40.24	0.694	38	23.03	271	46.40	<0.001 *
My child may get sick	55	10.93	28	11.38	0.855	31	18.79	52	8.90	<0.001 *
**Practice**										
Actions taken to get protected against Zika virus										
Using mosquito nets for protection	269	53.48	148	60.16	0.084	72	43.64	345	59.08	<0.001 *
Applying mosquito repellent to the skin or spreading anti-mosquito products	315	62.62	134	54.47	0.032 *	114	69.09	335	57.36	0.007 *
Wearing clothes that cover most of the body	205	40.76	94	38.21	0.504	89	53.94	210	35.96	<0.001 *
Using condoms or requiring partner to use condoms in every sexual encounter	54	10.74	40	16.26	0.032 *	21	12.73	73	12.50	0.938
Using modern methods for family planning	7	1.39	8	3.25	0.088	0	0.00	15	2.57	0.038 *
Abstaining from sex	17	3.38	13	5.28	0.212	1	0.61	29	4.97	0.012 *
Removing any pools of standing water	320	63.62	141	57.32	0.096	124	75.15	337	57.71	<0.001 *
Placing mosquito nets on windows and doors	180	35.79	90	36.59	0.830	82	49.70	188	32.19	<0.001 *
Actions to be taken if a person suspects he or she is infected with Zika										
Staying at home and doing nothing/not taking any medicine	47	9.34	35	14.23	0.044 *	9	5.45	73	12.50	0.010 *
Coming into the corresponding health center	445	88.47	210	85.37	0.229	154	93.33	501	85.79	0.010 *
Going to the drug store	22	4.37	21	8.54	0.021 *	5	3.03	38	6.51	0.127
Knowledge about there is no Zika vaccine	22	4.37	18	7.32	0.253	2	1.21	38	6.51	<0.001 *
Wish to receive more information about Zika	402	79.92	177	71.95	0.037 *	127	76.97	452	77.40	0.440
Desired aspects to receive more information										
Cause	279	55.47	148	60.16	0.223	83	50.30	344	58.90	0.049 *

* Refers to findings significant at *p* < 0.05.

**Table 3:** Predictors of general knowledge about Zika by applying both univariate and multivariate analyzes.

Variables/Categories	Low Level of Knowledge	Medium or High Level of Knowledge about Zika	Univariate Analysis		Multivariate Analysis	
	*n* (%)	*n* (%)	OR (CI 95)	*p*	OR (CI 95)	*p*
Sex ^a^						
Woman	42 (8.3)	461 (91.7)	0.771 (0.461–1.290)	0.321		
Man	26 (10.6)	220 (89.4)	1.0			
Status						
Workers	11 (6.7)	154 (93.3)	0.660 (0.338–1.291)	0.225		
Students	57 (9.8)	527 (90.2)	1.0			
Age Groups ^a^						
15–19	26 (9.9)	236 (90.1)	1.082 (0.516–2.269)	0.835		
20–24	28 (10.1)	249 (89.9)	1.104 (0.53–2.298)	0.791		
25–39	3 (3.3)	88 (96.7)	0.335 (0.091–1.237)	0.101		
40 and over	11 (9.2)	108 (90.8)	1.0			
Highest level of education ^a^						
High school or less	37 (10.3)	321 (89.7)	1.815 (0.823–4.006)	0.823		
Bachelor’s degree	22 (9.1)	219 (90.9)	1.582 (0.684–3.659)	0.684		
Posgraduate degree	8 (6.0)	126 (94.0)	1.0			
Academic Area ^a^	3 (2.5)	115 (97.5)				
Health Sciences	65 (10.3)	566 (89.7)	0.227 (0.070–0.735)	0.013	0.234 (0.071–0.778)	0.018 *
Others			1.0		1.0	
Region ^a^						
Coatzacoalcos-Minatitlán	8 (11.0)	65 (89.0)	2.113 (0.701–6.367)	0.184	2.6 (0.90**–**7.90)	0.092
Córdoba-Orizaba	7 (6.2)	106 (93.8)	1.134 (0.369–3.487)	0.827	1.6 (0.5**–**5.0)	0.414
Poza Rica-Tuxpan	22 (15.2)	123 (84.8)	3.07 (1.2–7.859)	0.019	3.1 (1.2–7.9)	0.019 *
Veracruz	13 (7.3)	165 (92.7)	1.353 (0.498–3.67)	0.553	1.5 (0.5–4.0)	0.45
Xalapa	12 (9.2)	119 (90.8)	1.731 (0.627–4.776)	0.289	2.0 (0.7–5.6)	0.176
No specified	6 (5.5)	103 (94.5)	1.0		1.0	

^a^ Data are presented in *n* (%); * Refers to findings significant at *p* < 0.05.

**Table 4:** Participant’s level of knowledge and practice when considering sex and status.

Characteristic	General	Sex		*p*	Status		*p*
		Women	Men		Students	Workers	
	*n* = 749	*n* = 503	*n* = 246		*n* = 584	*n* = 165	
Level of knowledge ^a^				0.080			0.469
Low	68 (9.1)	42 (8.3)	26 (10.6)		57 (9.8)	11 (6.7)	
Medium	595 (79.4)	411 (81.7)	184 (74.8)		461 (78.9)	134 (81.2)	
High	86 (11.5)	50 (10.0)	36 (14.6)		66 (11.3)	20 (12.1)	
Level of practice ^a^				0.748			0.140
Low	412 (55.0)	276 (54.9)	136 (55.3)		332 (56.8)	80 (48.5)	
Medium	306 (40.9)	208 (41.3)	98 (39.8)		230 (39.4)	76 (46.0)	
High	31 (4.1)	19 (3.8)	12 (4.9)		22 (3.8)	9 (5.5)	

^a^ Data are presented in *n* (%). An X^2^ test was carried out.

**Table 5:** Relationship between participant’s level of knowledge and practice when considering sex and status.

Level of Knowledge	Practice			*p*
	Low	Medium	High	
	*n* = 412	*n* = 306	*n* = 31	
				**<0.001**
Low	57 (13.8)	11 (3.6)	0 (0.0)	
Medium	336 (81.6)	244 (79.7)	15 (48.4)	
High	19 (4.6)	51 (16.7)	16 (51.6)	

An X^2^ test was carried out.
